# Intellectual disability and autism in propionic acidemia: a biomarker-behavioral investigation implicating dysregulated mitochondrial biology

**DOI:** 10.1038/s41380-023-02385-5

**Published:** 2024-01-11

**Authors:** Oleg A. Shchelochkov, Cristan A. Farmer, Colby Chlebowski, Dee Adedipe, Susan Ferry, Irini Manoli, Alexandra Pass, Samantha McCoy, Carol Van Ryzin, Jennifer Sloan, Audrey Thurm, Charles P. Venditti

**Affiliations:** 1 Organic Acid Research Section, National Human Genome Research Institute, National Institutes of Health, Bethesda, MD 20892 USA; 2 Neurodevelopmental and Behavioral Phenotyping Service, National Institute of Mental Health, National Institutes of Health, Bethesda, MD 20892 USA

**Keywords:** Psychology, Diagnostic markers, Biochemistry

## Abstract

Propionic acidemia (PA) is an autosomal recessive condition (OMIM #606054), wherein pathogenic variants in *PCCA* and *PCCB* impair the activity of propionyl-CoA carboxylase. PA is associated with neurodevelopmental disorders, including intellectual disability (ID) and autism spectrum disorder (ASD); however, the correlates and mechanisms of these outcomes remain unknown. Using data from a subset of participants with PA enrolled in a dedicated natural history study (*n* = 33), we explored associations between neurodevelopmental phenotypes and laboratory parameters. Twenty (61%) participants received an ID diagnosis, and 12 of the 31 (39%) who were fully evaluated received the diagnosis of ASD. A diagnosis of ID, lower full-scale IQ (sample mean = 65 ± 26), and lower adaptive behavior composite scores (sample mean = 67 ± 23) were associated with several biomarkers. Higher concentrations of plasma propionylcarnitine, plasma total 2-methylcitrate, serum erythropoietin, and mitochondrial biomarkers plasma FGF21 and GDF15 were associated with a more severe ID profile. Reduced 1-^13^C-propionate oxidative capacity and decreased levels of plasma and urinary glutamine were also associated with a more severe ID profile. Only two parameters, increased serum erythropoietin and decreased plasma glutamine, were associated with ASD. Plasma glycine, one of the defining features of PA, was not meaningfully associated with either ID or ASD. Thus, while both ID and ASD were commonly observed in our PA cohort, only ID was robustly associated with metabolic parameters. Our results suggest that disease severity and associated mitochondrial dysfunction may play a role in CNS complications of PA and identify potential biomarkers and candidate surrogate endpoints.

## Introduction

Propionic acidemia (PA) is an autosomal recessive disorder (OMIM #606054) caused by pathogenic variants in *PCCA* or *PCCB* which encode the subunits of the mitochondrial localized enzyme propionyl-CoA carboxylase (PCC). A deficiency of PCC leads to the accumulation of toxic metabolites (e.g., propionylcarnitine and 2-methylcitrate), disruption of mitochondrial metabolism, and dysregulation of signaling pathways [1]. PA carries a significant risk of mortality and age-dependent morbidity [1–3]. Common chronic symptoms include intellectual disability, epilepsy, movement disorders, sensorineural hearing loss, optic nerve atrophy, anemia, neutropenia, thrombocytopenia, cardiomyopathy, and chronic kidney disease. In severe forms, the clinical course is often punctuated by ketoacidotic and/or hyperammonemic metabolic crises resulting in acute encephalopathy. In mild cases, individuals with PA often remain metabolically stable, but may instead experience learning disabilities and autism spectrum disorder, in addition to dilated cardiomyopathy and chronic kidney disease [1–5].

The estimated birth incidence of PA varies by region, ranging 1:1000–1:500,000 worldwide, and 1:105,000–1:500,000 in the United States [6–9]. Although a rare metabolic disorder, PA has been recognized since the 1960s and is representative of a group of disorders termed organic acidemias, which are collectively common and difficult to treat inborn errors of metabolism.

One of the long term complications in individuals with PA is the poor cognitive and developmental outcomes [1–3]. Intellectual disability (ID) is common in this population; the largest retrospective case series found that 76% of 40 participants with IQ testing had scores in the range of ID [10], and a large registry survey reported developmental delays and/or deficits in cognitive, motor, and language skills in more than 70% (*N* = 40) [11]. Autism spectrum disorder (ASD) has only recently been assessed in studies of individuals with PA [12–14], and while common, it is usually observed in addition to ID. A recent registry study reported a history of ASD diagnosis among 10 of 48 respondents, the majority (*n* = 7) of whom also reported ID [14]. One study reported that among eight consecutive participants aged 3–21 years, all exhibited symptoms of ASD and five met criteria for an ASD diagnosis [13]. All but one of the eight participants also had mild-to-moderate ID. A later study documented symptoms of ASD in nine of 19 participants aged 2–25 years, four of whom met criteria for ASD diagnosis. Each participant with ASD also had mild-to-moderate ID [12]. Although progress has been made to document the prevalence of ID and ASD in PA, risk factors associated with neurodevelopmental outcomes remain largely unexplored, and predictive or associated biomarkers are unknown.

ID and ASD in PA are likely multifactorial in etiology. However, there have been few attempts to link them to the biochemical manifestations of PA. Previous work demonstrated that the number of metabolic crises experienced was inversely related to IQ (*N* = 40) [10], but data on specific biomarkers remain inconclusive. The aforementioned studies of ASD in individuals with PA explored a limited number of biochemical features: one study observed no associations between several metabolic markers (propionylcarnitine, 2-methycitrate, and urinary 3-OH-propionate) and an ASD diagnosis (*N* = 8) [13]. A larger study (*N* = 19) also found no relationship between urinary 3-OH-propionate and ASD, though higher levels were observed in the subset of the sample with ID [12]. This finding was in agreement with earlier work showing that among nine participants with ID, urinary 3-OH-propionate was higher among those with poorer neurocognitive outcomes [15].

Amino acids have also attracted significant attention as biomarkers associated with poor neurocognitive outcomes in individuals with inborn errors of metabolism. For example, in the context of urea cycle disorders, higher levels of plasma glutamine and ammonia correlated with lower neuropsychological test scores [16, 17]. Although elevated ammonia and abnormal plasma glutamine levels are common in individuals affected by PA, they were not associated with IQ [15]. Moreover, frequent observations of paradoxical hypoglutaminemia, especially during hyperammonemic metabolic crises, further confounds the ability to interpret the predictive role of ammonia and glutamine in the cognitive outcomes of individuals with PA, and more generally, organic acidemias. Surprisingly, hyperglycinemia, one of the defining biochemical features of PA, did not correlate with cognitive outcomes in at least one study [15].

More recently, FGF21 and GDF15, proteins associated with mitochondrial dysfunction, have been identified as potential response biomarkers associated with clinical outcomes of PA [4, 18, 19]. Plasma FGF21, a member of the FGF superfamily of proteins inducible by cellular stress, is elevated in mitochondrial disorders and organic acidemias [4, 18–22]. Plasma GDF15, a secreted protein linked to mitochondrial homeostasis, can be elevated in mitochondrial disorders and tracks with the severity of propionic acidemia [4, 23]. Other authors have hypothesized that GDF15 may also be associated with symptoms of ASD [13]. FGF21 and GDF15 might reflect the mitochondrial status in a wide range of disorders and thus hold promise to provide novel insights into the molecular mechanisms of PA [4].

In this study, we relied upon a unique resource, a well-characterized cohort of deeply phenotyped participants with PA in a natural history study conducted at the NIH Clinical Center, to explore the correlation between biochemical parameters and neurodevelopmental phenotypes to gain novel insights into ASD and ID in individuals with PA. The goal of this analysis was to identify a panel of predictive biomarkers that may be useful for prognostication and to assess the effects of interventions on the neurological phenotypes.

## Participants and methods

### Participants

The convenience sample of participants (*n* = 33) were ascertained via a longitudinal natural history study of individuals with PA (NCT02890342; protocol 16-HG-0156), and 31 were co-enrolled in a neurodevelopmental phenotyping study (NCT00271622; protocol 06-M-0065). Select data from this cohort have been published elsewhere [4, 5]. Informed consent and assent (when appropriate) were obtained from all participants and their guardians; study procedures were approved by the NIH Institutional Review Board. Inclusion criteria for the natural history study were age 2 years or older, with a diagnosis of PA confirmed biochemically and/or molecularly (Table S1). Exclusion criteria were poor metabolic control, lack of a medical genetic provider, and intercurrent infection. Participants were included in the current analysis if they contemporaneously completed at least one of the neurodevelopmental assessments and contributed a blood and/or urine sample. They were excluded from the current analysis if they had undergone an organ transplant at any point prior to the study visit. Some participants had multiple visits which met these criteria; in that case, the visit with the most complete data was selected.

### Procedures

#### Clinical, biochemical and molecular studies

The clinical variables included in this study were identified either through the review of literature or by a previous analysis of ~500 variables from the full NIH PA cohort [4]. Concurrent blood and/or urine samples were collected from all participants during an inpatient visit with plasma propionylcarnitine, glycine, glutamine, serum erythropoietin, and urinary glutamine obtained per protocol. Plasma 2-methylcitrate was measured using a gas chromatographic-mass spectrometric assay [24]. For six participants, the reference lab reported plasma propionylcarnitine levels as “> 60 µmol/L,” which was entered in the dataset as 60 µmol/L. FGF21 was measured using Human FGF21 Quantikine ELISA (R&D System, USA). GDF15 was measured either using Human GDF15 Quantikine ELISA (R&D System, USA) or by a reference lab (Mayo Clinic, Rochester, MN; package insert: Human GDF15 Immunoassay. R&D Systems; 2014).

The in vivo stable isotope study using oxidation of 1-^13^C-propionate to ^13^CO_2_ was conducted as previously described [4, 19]. Briefly, baseline CO_2_ production (V_CO2_) was measured with indirect calorimetry [25]. 1-^13^C-propionate was given either orally or as a bolus via enteral tube. Breath samples of ^13^CO_2_ were collected serially. Isotope ratio mass spectrometry (^13^C/^12^C) of CO_2_ in the expired breath was used to calculate the percent ^13^CO_2_ recovery over 2 h (at 30, 60, 120 min intervals). Baseline CO_2_ production rate was used to calculate propionate oxidation capacity [25, 26]. For one participant, propionate oxidation was assessed 15 months prior to the neurodevelopmental assessment; this subject did not contribute a blood or urine sample. All other 1-^13^C-propionate oxidation assessments occurred contemporaneously with the neuropsychological evaluation.

#### Neurodevelopmental assessments

The neurodevelopmental assessment battery was completed over the course of 1–2 days within 1 month of the blood sample. A team of doctoral-level psychologists performed the neurodevelopmental evaluations assessing cognitive ability, adaptive skills, and the symptoms and diagnostic criteria for ASD as previously described [27].

Given the ranges of age and ability in this cohort, a hierarchy of cognitive tests was employed (age range in parentheses): Mullen Scales of Early Learning (birth–68 months), Differential Abilities Scales (30 months–17 years 11 months), Wechsler Preschool and Primary Scale of Intelligence (30 months–7 years 7 months), Weschler Intelligence Scale for Children (6 years–16 years 11 months), Wechsler Adult Intelligence Scale (16–90 years), Weschler Abbreviated Intelligence Scales (6–90 years). Consistent with common practice in studies involving ID [27], out-of-age-range testing was accommodated through use of ratio IQs. The full-scale IQ (FSIQ) (population mean = 100, standard deviation = 15) was used in the current study. The accommodations that the child typically used (e.g., hearing aids, glasses) were allowed.

Adaptive behavior was assessed with the parent interview form of the Vineland Adaptive Behavior Scales, Second Edition. The overall Adaptive Behavior Composite (ABC; population mean = 100, standard deviation = 15, floor = 20) was used in the current study.

The diagnosis of DSM-5 ID was made based on cognitive and adaptive behavior test results and clinical judgment. Participants were screened for ASD using records review, assessment of parental concern about ASD, and the Social Communication Questionnaire [28]. If the participant did not screen positively, ASD was ruled out. When screening indicated concern(s), the participant received a comprehensive ASD assessment battery conducted by doctoral-level clinical psychologists with research reliability in the Autism Diagnostic Observation Schedule, Second Edition and the Autism Diagnostic Interview-Revised. Ultimately, a research diagnosis of ASD based on DSM-5 criteria was made by clinical consensus using all available information, including ADOS-2 and ADI-R scores.

#### Statistical analysis

Statistical analysis was performed using R version 4.2.1. Phenotypic features were summarized using descriptive statistics. We constructed a series of linear models predicting a given neuropsychological feature from a given biomarker. For continuous outcomes, the expected distribution was normal and visual inspection of residual plots was used to guide evaluation of the associated assumptions. For the categorical diagnostic outcomes, the expected distribution was binomial with a logit link function. In the cases of quasi-complete separation, Firth logistic regression was substituted. To improve interpretability of coefficients, biomarker values were natural log-transformed. Because the effect of the biomarkers on the outcomes may be confounded by age [4, 5], age was entered as a covariate in all models. The sample size of this study precluded the evaluation of interaction terms between age and the biomarkers. The parameter of interest was the slope of the biomarker on the outcome; for the logistic models this was expressed as an odds ratio. In keeping with the recommendations of the American Statistical Association [29], we report uncorrected p-values alongside parameter estimates and their 95% confidence intervals. Where additional qualitative descriptions of these parameter estimates (e.g., strong) are given, they are subjective evaluations based on our clinical knowledge of the measurement and distributional properties of the outcome measure of interest. Of note, there was one set of twins in the dataset; analyses were not adjusted for this fact.

## Results

Thirty-three participants met inclusion criteria for the current analysis. Participant demographics are summarized in Table 1. Participants ranged in age from 2 to 38 years (median=16, IQR = 8–24). Biallelic *PCCB* pathogenic variants were most prevalent (*n* = 20, 61%; genetic characteristics are detailed in Table S1). Mild-to-severe sensorineural hearing loss was documented for *n* = 13/28 (46% of those with available data), and optic nerve abnormality or atrophy of various degree was documented for *n* = 8/30 (27% of those with available data).

**Table 1:** Sample Characteristics.

Descriptor	*n* (%)
Age	
Years, M(SD)	16.67 (10.43)
< 18 years	21 (64%)
Female sex	18 (55%)
Race	
Black or African American	4 (12%)
Multiple races	2 (6%)
Unknown	1 (3%)
White	26 (79%)
Ethnicity	
Latino or Hispanic	4 (12%)
Not Latino or Hispanic	27 (82%)
Missing	2 (6%)
Optic nerve abnormality	
No	22 (67%)
Yes	8 (24%)
Unknown	3 (9%)
Hearing loss	
No	15 (45%)
Yes	13 (39%)
Unknown	5 (15%)

### Variable severity of PA neurodevelopmental profile

Neurodevelopmental data for the sample are summarized in Table 2. Individual results are provided in Table S2. There was a broad range of FSIQ and ABC scores, 14–109 and 20–108, respectively. The mean scores on both outcomes (FSIQ: 65 ± 26, ABC: 67 ± 23) were more than two standard deviations away from the mean ( < 2nd percentile). The correlation between FSIQ and ABC was *r* = 0.74.

**Table 2:** Summary of genetic, biochemical, and neurodevelopmental features.

Descriptor	*n* (%)	Mean (SD)	Median (IQR)	Range
Gene Affected				
*PCCA*	13 (39%)			
*PCCB*	20 (61%)			
Two loss-of-function (LOF) alleles				
Yes	10 (30%)			
No	23 (70%)			
Biomarkers				
1-^13^C-Propionate Oxidation, %	30 (91%)	8.73 (9.19)	4.27 (1.87–15.38)	0.39–32.04
Propionylcarnitine, µmol/L	32 (97%)	48.79 (27.63)	43.71 (29.19–60)	11.73–154.71
Erythropoietin, mIU/mL	28 (85%)	13.09 (5.69)	12.75 (8.38–16.6)	3.8–25.4
FGF21, pg/mL	32 (97%)	2201.87 (2671.43)	1329.26 (555.14–2645.5)	48.11–11890.59
GDF15, pg/mL	32 (97%)	1453.38 (1270.1)	940.93 (544.87–1989.5)	162.35–4691.72
Plasma glutamine, µmol/L	32 (97%)	588.12 (171.56)	598.5 (483.5–665.75)	273–959
Urine glutamine, nmol/mg creatinine	27 (82%)	447.63 (339.53)	347 (158.5–600)	85–1217
Glycine, µmol/L	32 (97%)	811.41 (408.41)	755 (518.5–1009.25)	248–1909
Total 2-methylcitrate, nmol/L	32 (97%)	40292.09 (47288.33)	21591.5 (15797–47973.25)	3167–230675
Neurocognitive Outcomes				
Full Scale IQ^1^	33 (100%)	64.76 (25.72)	62 (49–85)	13.75–109
Adaptive Behavior Composite^2^	31 (94%)	66.9 (23.31)	65 (53–87)	20–108
Autism Spectrum Disorder (ASD) Diagnosis				
No	19 (58%)			
Yes	12 (36%)			
Unknown	2 (6%)			
Intellectual Disability (ID) Diagnosis				
No	13 (39%)			
Yes	20 (61%)			
Combined ASD and ID (*n* = 31)				
ASD + ID	9 (29%)			
ASD only	3 (10%)			
ID only	10 (32%)			
Neither	9 (29%)			

^1^See Supplementary Table S2 for test used.

^2^One participant received the Vineland-3; the remainder received the Vineland-II.

The diagnosis of ID was made in 20 (61%; eight male) participants, including a diagnosis of global developmental delay that was used for one participant who was too young to meet criteria for ID. Of the 31 participants for whom a determination could be made (see Fig. S1), 12 (39%; six male) received a diagnosis of ASD. The mean FSIQ and ABC scores were lower among those with an ASD diagnosis (FSIQ: 53 ± 25, ABC: 54 ± 23) than for those without (FSIQ: 73 ± 25, ABC: 75 ± 20). Compared to participants without ASD, those with ASD were more likely to also have ID (10/19 versus 9/12, corresponding to an OR of 2.7).

### Neurodevelopmental outcomes are associated with PA parameters

**Fig. 1:**
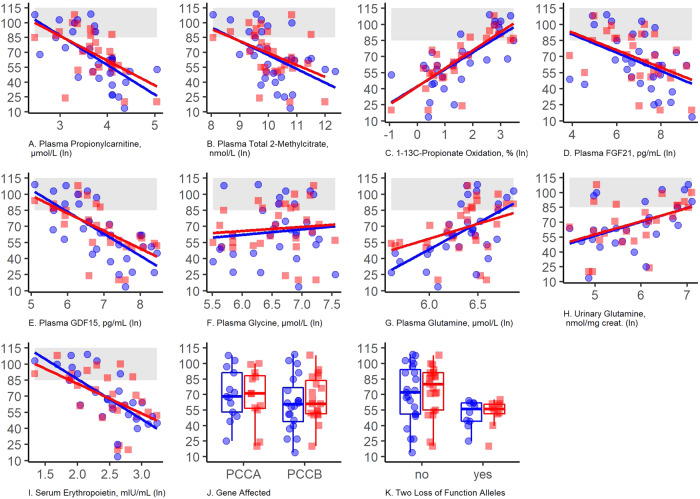
Neurocognitive outcomes in PA are associated with biomarkers linked to the mitochondrial dysfunction and the severity of PCC deficiency. Biomarkers shown here as *natural log-transformed* values were linked via regression to the underlying severity of PA and mitochondrial dysfunction and showed similar associations with full scale IQ and Vineland Adaptive Behavior Composite scores. Red and blue lines illustrate an unadjusted linear regression for the outcomes (Y-axis) full scale IQ (blue) and Vineland Adaptive Behavior Composite (red). The log-transformed predictor variable for each panel is named in the header. The population-average standard score range (85–115) is shaded in gray. Parameter estimates corresponding to these lines are shown in Table 3 and test statistics in Supplementary Table S3. The distributions of *untransformed* continuous biomarkers are shown in Supplemental Figure S2. **A** Plasma propionylcarnitine (ln-transformed). **B** Plasma total 2-methylcitrate (ln-transformed). **C** In vivo whole body 1-^13^C-propionate oxidation (ln-transformed). **D** Plasma FGF21 (ln-transformed). **E** Plasma GDF15 (ln-transformed). **F** Plasma glycine (ln-transformed). **G** Plasma glutamine (ln-transformed). **H** Urinary concentration of glutamine normalized by creatinine (ln-transformed). **I** Serum erythropoietin (ln-transformed). **J** Affected gene, *PCCA vs PCCB*. **K** Comparison of neurocognitive outcomes in participants with two (biallelic) loss-of-function *PCCA* or *PCCB* alleles vs all other genotypes (e.g., two missense alleles or one missense plus one nonsense allele).

Descriptive figures illustrating the relationship of PA mutations and biomarkers to diagnostic outcomes are found in Fig. 1, and regression results are shown in Table 3 (see Fig. S2 for distributional plots). Whether an individual had pathogenic variants in *PCCA* or *PCCB* was not associated with FSIQ or ABC scores, but participants with biallelic pathogenic variants in *PCCB* had elevated odds of an ID diagnosis relative to those with variants in *PCCA* (observed probability 75% versus 38%; OR = 5.5[1.2, 30]). All participants (100% of *n* = 10) harboring two loss-of-function alleles in either *PCCA* or *PCCB* (nonsense, deletions, copy number variants or variants affecting canonical splice sites) were diagnosed with ID, compared to 10 of 23 (43%) with any other combination of alleles (OR not calculated because of complete separation). The rate of two loss-of-function alleles was similar between participants with pathogenic variants in *PCCB* (6/20, 30%) and *PCCA* (4/13, 31%). Among those who did not have two loss-of-function alleles, nine of 14 (64%) participants with *PCCB* mutations had ID versus only one of nine (11%) in those with *PCCA* mutations. In the full sample, the presence of two loss-of-function alleles was associated with lower FSIQ (B = −19 [−39, 0.5] and ABC (B = −19 [−36, −2]).

**Table 3:** Coefficients of regression models associated with diagnostic outcomes, adjusting for age.

Predictor	ASD Diagnosis	ID Diagnosis	FSIQ	ABC
	OR [95% CI]	OR [95% CI]	B [95% CI]	B [95% CI]
PCCB gene^1^	0.26 [0.05, 1.18]	5.50 [1.21, 30.10]	−9.85 [−29.10, 9.40]	−4.12 [−21.49, 13.26]
Two loss-of-function alleles^2^	3.85 [0.76, 22.83]	NA^3^	−19.2 [−38.94, 0.54]	−18.98 [−36.36, −1.6]
1-^13^C-Propionate oxidation, 60-min, % (ln)	0.57 [0.25, 1.18]	0.04 [ < 0.001, 0.24]^4^	15.72 [9.69, 21.76]	15.9 [10.89, 20.9]
Propionylcarnitine, plasma, µmol/L (ln)	1.88 [0.41, 10.55]	2203.06 [32.13, 8436433.3]	−30.18 [−43.78, −16.59]	−23.46 [−38.45, −8.46]
Erythropoietin, serum, mIU/mL (ln)	28.11 [2.55, 1050.72]	115.39 [6.82, 10245.56]	−36.77 [−52.62, −20.92]	−25.12 [−43.23, −7.01]
FGF21, plasma, pg/mL (ln)	0.79 [0.43, 1.38]	2.01 [1.1, 4.31]	−8.52 [−15.28, −1.77]	−7.88 [−13.58, −2.18]
GDF15, plasma, pg/mL (ln)	1.51 [0.62, 3.99]	59.83 [6.59, 2743.82]	−22.23 [−30.01, −14.45]	−15.92 [−24.28,−7.57]
Glutamine, plasma, µmol/L (ln)	0.07 [0, 0.9]	0.01 [0, 0.18]	51.07 [26.09, 76.05]	31.78 [7.47, 56.1]
Glutamine, urine, nmol/mg creatinine (ln)	0.48 [0.1, 2.07]	0.03 [0, 0.26]	17.88 [3.75, 32.01]	11.23 [−5.68, 28.13]
Glycine, plasma, µmol/L (ln)	0.85 [0.21, 3.31]	0.69 [0.17, 2.57]	5.59 [−12.44, 23.63]	6.05 [−9.42, 21.52]
Total 2-methylcitrate, plasma, nmol/L (ln)	1.36 [0.46, 4.44]	91.81 [7.41, 7176.88]	−16.11 [−26.26, −5.95]	−11.23 [−23.03, 0.58]

^1^Reference is *PCCA* gene

^2^Reference is < 2 loss-of-function alleles

^3^All individuals with two loss-of-function alleles (*n* = 10) had a diagnosis of ID, compared to 10 of 23 in those with fewer than two loss-of-function alleles. Due to quasi-complete separation, computation of meaningful statistics was not possible.

^4^Quasi-complete separation occurred because the distributions of propionate oxidation were almost non-overlapping between those with and without ID. Firth’s logistic regression was used for this analysis.

*ASD* Autism spectrum disorder, *ID* Intellectual disability, *FSIQ* Full scale intelligence quotient, *ABC* Vineland adaptive behavior composite. All models contained age as a covariate. Continuous predictors were natural-log transformed, indicated by (ln). OR > 1 indicates that increased value of variable is associated with increased odds of diagnosis; OR < 1 indicate that increased value of variable is associated with decreased odds of diagnosis. Test statistics and exact p-values are found in Supplementary Table S3.

Specific genotypes were not robustly associated with ASD diagnosis, though participants with *PCCA* were more likely than those with *PCCB* to have an ASD diagnosis (observed probability 7 of 12, 58% versus 5 of 19, 26%; OR = 0.26[0.50, 1.18]).

Several biomarkers were associated with both an ID diagnosis and FSIQ and ABC scores (Table 3). Increased levels of plasma propionylcarnitine, FGF21, GDF15, total 2-methylcitrate, and serum erythropoietin were strongly associated with lower FSIQ and ABC, and all 95% confidence intervals excluded zero. Conversely, decreased in vivo 1-^13^C-propionate oxidation and both plasma and urinary glutamine were associated with lower FSIQ and ABC. As illustrated in Fig. 1 and by the overlapping confidence intervals of the regression slopes (Table 3), relationships between a given biomarker and FSIQ and ABC were very similar. Only two biomarkers, increased serum erythropoietin and decreased plasma glutamine, were strongly associated with ASD diagnosis, with 95% CI excluding zero (Table 3) (see also Fig. S3). Plasma glycine, one of the defining biochemical features of PA, was not meaningfully associated with any neurodevelopmental outcome.

## Discussion

This cross-sectional PA cohort is drawn from the largest prospective single-center natural history study of participants with PA and has enabled characterization of long-term sequelae, the discovery of novel candidate PA biomarkers, and their associations with outcomes [4, 5]. In the current work, we first confirmed previous observations that PA is associated with the neurodevelopmental disorders ID and ASD. Consistent with extant work [10, 12–14], the majority (61%) of the NIH sample met criteria for an ID diagnosis, a significant proportion (39%) met rigorously applied diagnostic criteria for ASD, and most (75%) of those who met criteria for ASD also met criteria for ID. The remarkably high prevalence of ASD that has now been consistently observed in studies of PA is common across a variety of monogenic conditions. Examples include fragile X syndrome ( ~ 50%), tuberous sclerosis complex (26–45%), *PACS1*-related neurodevelopmental disorder (25–30%), and Smith-Lemli-Opitz syndrome (55–71%) [30–33]. In these rare genetic conditions, too, a high rate of comorbidity with ID and a lack of sex differences in rate of ASD diagnosis is observed [34]. This subgroup of monozygotic conditions featuring high prevalence of ID with co-existing ASD creates an opportunity to explore biological insights into molecular mechanisms shared by, or differentiating between, the given neurodevelopmental disorders.

In this cohort [13], we did not detect a robust association between the *PCCA* and *PCCB* alleles and the ASD diagnosis, though there was a trend for those with *PCCA* (58%) to be more likely to be diagnosed than those with *PCCB* (26%). We demonstrated that both FSIQ and adaptive behavior scores are strongly associated with plasma propionylcarnitine, total 2-methylcitrate, FGF21 and GDF15 [35, 36] as well as 1-^13^C-propionate oxidation which relies upon the in vivo metabolism of labelled propionate as readout for the residual enzymatic activity of PCC. All five of these parameters have been previously shown to be associated with the biochemical and clinical severity in propionic acidemia and have the potential to identify individuals at higher risk for adverse neurocognitive outcomes and prompt adjustments to clinical intervention [4].

We also discovered that those with lower levels of plasma glutamine and urinary glutamine were more likely to receive an ID diagnosis and had more impaired IQ and adaptive behavior scores. This association contrasts with the findings seen in urea cycle disorders, where higher plasma glutamine tends to be associated with higher plasma ammonia inversely related to neurocognitive outcomes [17, 37, 38]. Such an inverse relationship between plasma glutamine and plasma ammonia in PA has been reported previously [39]. It was hypothesized that low glutamine levels may reflect consumption of glutamine to replenish mitochondrial α-ketoglutarate. Thus, hypoglutaminemia appears to be a risk factor for lower FSIQ in those with PA, likely reflecting impaired mitochondrial function and TCA anaplerosis.

An intriguing finding arising from this study is the association of serum erythropoietin with ASD and ID diagnosis, as well as IQ and adaptive behavior scores. Along with plasma glutamine, serum erythropoietin was one of only two biomarkers in this cohort found to be associated with both ID and ASD diagnoses. Neurocognitive scores in this cohort with PA were inversely related to erythropoietin, i.e. the risk of ASD and ID was greater with higher levels of erythropoietin. None of the participants with PA had erythropoietin in the deficient range and in several severe affected participants, erythropoietin was elevated. Although the association between neurocognitive outcomes and erythropoietin could be a fortuitous discovery, we note that erythropoietin garnered interest for its role in reducing apoptosis, excitotoxicity, inflammation, and oxidative stress potentially associated with improved outcomes in pre-clinical studies of neonatal encephalopathy [40]. It has also been identified as a neuromodulating agent able to regulate mitochondrial function in the hippocampus and affect cognitive outcomes in an animal model, and that *EP300*, a transcriptional regulator of EPO, appears on a “high-confidence” list of genes associated with neurodevelopmental disorders [41–44]. A recent study highlighted a potential role of erythropoietin in improving autistic-like behaviors in rats with the valproate-induced model of autism, associated with the restored expression of K-Cl cotransporter encoded by *Slc12a5* [45]. Another study suggested that an association may exist between high umbilical cord serum EPO levels and an increased risk for severe neurocognitive morbidity in early childhood even after adjusting for the gestational age [46]. Furthermore, because erythropoietin levels did not correlate with the hematocrit or estimated glomerular filtration rate (data not shown) in the participants studied here, the association between elevated erythropoietin levels and an ASD diagnosis appears unrelated related to chronic kidney disease, a common complication of PA [5], and we believe likely reflects an additional biological role for the erythropoietin axis in CNS pathology.

We believe that the association identified in this study between propionic acid derivatives (e.g., propionylcarnitine, 2-methylcitrate) and adverse neurocognitive outcomes is specific to PA. Concentrations of PA-related metabolites in PA are many orders of magnitude greater than in controls, therefore, chronic exposures to endogenously produced metabolites is a unique biochemical state typically not observed under physiological conditions. Similarly, the elevations in GDF15, FGF21, EPO, and glutamine, in the setting of increased propionyl-CoA derived metabolites, is unique. While these variables can be associated with ID and/or ASD in PA, they will almost certainly lack their positive predictive power in other clinical contexts. However, what connects PA-related ID and ASD to similar outcomes in other diseases is their convergence on impaired mitochondrial biology. It has been hypothesized that impaired mitochondrial function can explain many symptoms seen in autism [47–49]. In this aspect, our work adds more evidence in support of the mitochondrial hypothesis of neurocognitive pathology and expands the repertoire of biological factors pointing to its possible mechanisms.

While there was significant overlap in the diagnosis of ID and ASD in this sample, it was not complete. ASD was diagnosed in some participants who did not have ID and vice versa. Only 53% of those diagnosed with ID were diagnosed with ASD, and 67% of those diagnosed with ASD had ID. That the biomarkers were more consistently associated with ID than ASD prompted a revisitation of the debate about the existence of pathways showing stronger associations with ID than ASD [50–53]. We considered several potential factors (not mutually exclusive) that could produce divergence among risk factors including limitations of testing and the effect of small dataset size, temporal factors not captured by the cross-sectional subset of NIH PA cohort, and the differential response of neuronal circuits underwriting intellectual functions and social interactions. While there are no specific measures of autism symptom severity, available measures such as the Social Responsiveness Scale are confounded by low cognitive and language ability [54, 55]. The lack of specificity is especially significant in our patient cohort, where a very high rate of overlap was observed in ID and ASD diagnoses. Because the cross-sectional nature of our dataset limits our ability to assess the role of temporal relationships, such as the effect of hyperammonemia, the number of metabolic decompensations, age of symptom onset, hearing loss, dietary changes, and vision impairment, we are unable to ascertain causality between biomarkers and neurodevelopmental disorders. For participants with a variable onset of treatment (e.g., those not detected by newborn screening), it is difficult to establish the role of any biomarker in early development without prospective study. In addition, it is possible that prolonged exposure of the brain to endogenously produced propionate, related metabolites, and/or associated mitochondrial dysfunction may have varied effects on brain connectomes underwriting ID and ASD [56, 57]. The associations identified in our study are based on correlational data and replication in independent cohorts, augmented by further mechanistic work in animal models, will be needed to understand the causal relationship between biomarkers described here and outcomes in PA. The associations in this study are based on correlational data and cannot be assumed to imply causation or therapeutic potential, and our study was underpowered to evaluate the effect of hyperammonemia, the number of metabolic decompensations, age of symptom onset, hearing loss, vision impairment, MRI findings, and diet on neurocognitive outcomes of PA. Studies described here were performed under conditions of metabolic homeostasis, therefore labs (e.g. plasma glutamine or propionylcarnitine) collected during acute decompensations cannot be used for stratification of PA outcomes. Furthermore, novel observations afforded by this study require replication in independent cohorts. Longitudinal observations and mechanistic work will be needed to understand the causal relationship between biomarkers described here and other outcomes.

In conclusion, the association of neurodevelopmental outcomes with parameters reflecting mitochondrial dysfunction and the severity of propionyl-CoA carboxylase deficiency (plasma propionylcarnitine, FGF21, GDF15, total 2-methylcitrate, 1-^13^C-propionate oxidation and both plasma and urinary glutamine) helps define PA as a bona fide neurometabolic disorder, consistent with the evolving view that dysregulated mitochondrial function is a risk factor for neurodevelopmental outcomes [48]. Our findings should be of interest to investigators focusing on the molecular underpinning of ID and ASD, and regulatory specialists looking to establish biomarkers and surrogate endpoint in the future clinical trials of PA.

### Supplementary information

Supplementary materials

## Data Availability

The data and code used to generate these results are available upon reasonable request to the corresponding author.
