# Supplementary material for: Intellectual disability and autism in propionic acidemia: a biomarker-behavioral investigation implicating dysregulated mitochondrial biology
Source: Mol Psychiatry. 2024 Jan 11;29(4):974–81. doi: 10.1038/s41380-023-02385-5 (PMC11176071; doi:10.1038/s41380-023-02385-5)

**Table S1. Genetic information for participants.** Participant order is determined by genotype and age (see Table S2).

| ID | Gene Affected | Variant 1, cDNA | Variant 1, predicted protein | Variant 2, cDNA | Variant 2, predicted protein | Two LOF alleles |
| --- | --- | --- | --- | --- | --- | --- |
| P1 | PCCA | c.1284+1G>A | p.? | c.1684T>C | p.Ser562Pro | no |
| P2 | PCCA | c.1284+1G>A | p.? | c.1684T>C | p.Ser562Pro | no |
| P3 | PCCA | c.802C>T | p.Arg268Cys | c.1899+4_1899+7del | p.? | no |
| P4 | PCCA | c.742G>A | p.Glu248Lys | c.1430G>T | p.Gly477Val | no |
| P5 | PCCA | exon 23-24 deletion | N/A | exon 23-24 deletion | N/A | yes |
| P6 | PCCA | c.893A>G | p.Lys298Arg | exon 13-20 deletion | N/A | no |
| P7 | PCCA | c.1284+1G>A | p.? | c.2027del | p.Lys676SerfsTer6 | yes |
| P8 | PCCA | c.1268C>T | p.Pro423Leu | c.1899+4_1899+7del | p.? | no |
| P9 | PCCA | c.866_867del | p.Glu289ValfsTer53 | c.2162_2163insAG | p.Asp722GlyfsTer32 | yes |
| P10 | PCCA | c.716+5G>C | p.? | c.782A>G | p.Glu261Gly | no |
| P11 | PCCA | c.600+1G>A | p.? | c.2119-9A>G | p.? | yes |
| P12 | PCCA | c.1572_1573del | p.Gln524HisfsTer29 | c.434T>C | p.Phe145Ser | no |
| P13 | PCCA | c.862A>G | p.Arg288Gly | c.1214C>G | p.Pro405Arg | no |
| P14 | PCCB | c.990dup | p.Glu331Ter | c.1225_1227del | p.Ile409del | no |
| P15 | PCCB | c.764-2del | p.? | c.975_977del | p.Asp325del | no |
| P16 | PCCB | c.764-2del | p.? | c.975_977del | p.Asp325del | no |
| P17 | PCCB | c.386_387delinsAAC | p.Phe129Ter | c.1606A>G | p.Asn536Asp | no |
| P18 | PCCB | c.683C>T | p.Pro228Leu | c.1218_1231delinsTAGAGCACAGGA | p.Gly407ArgfsTer14 | no |
| P19 | PCCB | c.866G>C | p.Arg289Pro | c.990dup | p.Glu331Ter | no |
| P20 | PCCB | c.683C>T | p.Pro228Leu | c.1218_1231delinsTAGAGCACAGGA | p.Gly407ArgfsTer14 | no |
| P21 | PCCB | c.337C>T | p.Arg113Ter | c.1225_1227del | p.Ile409del | no |
| P22 | PCCB | c.1606A>G | p.Asn536Asp | c.1606A>G | p.Asn536Asp | no |
| P23 | PCCB | c.1172_1173del | p.Phe391CysfsTer2 | c.1172_1173del | p.Phe391CysfsTer2 | yes |
| P24 | PCCB | c.386_387delinsAAC | p.Phe129Ter | c.1552del | p.Asp518ThrfsTer33 | yes |
| P25 | PCCB | c.1218_1231delinsTAGAGCACAGGA | p.Gly407ArgfsTer14 | c.990dup | p.Glu331Ter | yes |
| P26 | PCCB | c.1218_1231delinsTAGAGCACAGGA | p.Gly407ArgfsTer14 | c.1218_1231delinsTAGAGCACAGGA | p.Gly407ArgfsTer14 | yes |
| P27 | PCCB | c.1204del | p.Ala402HisfsTer41 | c.335G>A | p.Gly112Asp | no |
| P28 | PCCB | c.76dup | p.Arg26ProfsTer11 | c.1218_1231delinsTAGAGCACAGGA | p.Gly407ArgfsTer14 | yes |
| P29 | PCCB | c.990dup | p.Glu331Ter | c.975_977del | p.Asp325del | no |
| P30 | PCCB | c.1218_1231delinsTAGAGCACAGGA | p.Gly407ArgfsTer14 | c.967G>T | p.Val323Phe | no |
| P31 | PCCB | c.734G>A | p.Gly245Asp | exon 9 deletion | N/A | no |
| P32 | PCCB | c.1218_1231delinsTAGAGCACAGGA | p.Gly407ArgfsTer14 | c.1495C>T | p.Arg499Ter | yes |
| P33 | PCCB | c.1218_1231delinsTAGAGCACAGGA | p.Gly407ArgfsTer14 | c.1606A>G | p.Asn536Asp | no |

*PCCA* NCBI Reference Sequence: NM_000282.3. *PCCB* NCBI Reference Sequence: NM_000532.4.

**Table S2. Demographic, phenotypic, and neuropsychological data.** Sex: F = female, M = male. Race: B|AA = Black or African-American, Mult = Multiple, W = White. FSIQ Test: DAS = Differential Abilities Scale; MSEL = Mullen Scales of Early Learning; WAIS = Wechsler Abbreviated Intelligence Scale; WASI = Wechsler Abbreviated Scale of Intelligence; WISC = Wechsler Intelligence Scale for Children; WPPSI = Wechsler Preschool and Primary Scale of Intelligence.

| ID | Sex | Race | Latino or Hispanic | Assessment Age (years) | Hearing Loss | Optic Nerve Abnormality | ASD Diagnosis | ID Diagnosis | FSIQ Test | FSIQ Score | Vineland ABC | ADOS-2 Module | ADOS-2 Classification | ADI-R Classification |
| --- | --- | --- | --- | --- | --- | --- | --- | --- | --- | --- | --- | --- | --- | --- |
| P1 | F | W | No | 6.61 | No | No | No | No | WPPSI | 91 | 100 | N/A | N/A | N/A |
| P2 | M | W | No | 8.1 | No | No | Yes | No | WISC | 67 | 87 | 3 | Autism | No |
| P3 | M | Mult | Yes | 9.75 | No | No | Yes | No | WASI | 108 | 85 | 3 | Autism spectrum | Yes |
| P4 | F | W | No | 10.09 | No | No | No | No | WISC | 103 | 99 | N/A | N/A | N/A |
| P5 | F | W | No | 11.63 | Yes | Yes | Yes | Yes | DAS | 25 | 57 | 2 | Autism | Yes |
| P6 | F | W | No | 12.34 | Yes | No | Yes | Yes | WISC | 49 | 71 | 3 | Autism | Yes |
| P7 | F | W | No | 12.52 | No | Yes | Yes | Yes | WISC | 44 | 55 | 3 | Autism | Yes |
| P8 | M | N/A | Yes | 16.81 | No | No | No | No | WISC | 72 | 71 | N/A | N/A | No |
| P9 | F | W | No | 19.5 | Yes | Yes | No | Yes | WAIS | 62 | 59 | 4 | Autism spectrum | No |
| P10 | F | W | No | 26.21 | Yes | No | Yes | No | WAIS | 75 | 24 | 4 | Autism spectrum | Yes |
| P11 | M | W | No | 36.2 | Yes | Yes | Yes | Yes | WAIS | 53 | 20 | 3 | Autism | Yes |
| P12 | F | B\|AA | N/A | 37 | No | No | N/A | No | WAIS | 68 | N/A | N/A | N/A | N/A |
| P13 | M | W | No | 38.33 | Yes | No | No | No | WAIS | 97 | 91 | N/A | N/A | N/A |
| P14 | M | Mult | No | 2.37 | No | No | No | Yes | MSEL | 74 | 76 | T | Non-spectrum | N/A |
| P15 | M | W | No | 5.29 | N/A | No | No | Yes | MSEL | 37.02 | 55 | N/A | N/A | N/A |
| P16 | M | W | No | 5.29 | N/A | Yes | No | Yes | MSEL | 27.17 | 50 | N/A | N/A | N/A |
| P17 | M | W | No | 5.36 | No | No | No | No | WPPSI | 91 | 80 | 3 | Autism | No |
| P18 | F | W | No | 5.45 | No | No | No | No | WPPSI | 103 | 91 | N/A | N/A | N/A |
| P19 | F | B\|AA | No | 6 | Yes | No | No | Yes | WPPSI | 58 | 72 | N/A | N/A | N/A |
| P20 | F | W | No | 6.24 | No | No | No | No | WISC | 109 | 94 | 3 | Non-spectrum | No |
| P21 | M | W | No | 8.6 | N/A | N/A | Yes | Yes | MSEL | 27.11 | 54 | 1 | Autism | Yes |
| P22 | M | W | No | 10.86 | No | No | No | No | WISC | 85 | 87 | 3 | Non-spectrum | No |
| P23 | F | W | No | 16.08 | Yes | No | No | Yes | WISC | 62 | 61 | 3 | Non-spectrum | No |
| P24 | M | W | Yes | 16.58 | Yes | Yes | Yes | Yes | WISC | 62 | 61 | 3 | Autism | Yes |
| P25 | F | B\|AA | Yes | 17.56 | No | No | Yes | Yes | WAIS | 45 | 52 | 2 | Autism | Yes |
| P26 | F | W | No | 17.72 | Yes | No | No | Yes | WASI | 40 | 51 | N/A | N/A | N/A |
| P27 | M | W | No | 19.23 | Yes | Yes | Yes | Yes | MSEL | 13.75 | 20 | 1 | Autism spectrum | Yes |
| P28 | F | W | No | 21.35 | Yes | No | Yes | Yes | WAIS | 64 | 65 | 3 | Autism | Yes |
| P29 | F | B\|AA | N/A | 24 | No | N/A | N/A | Yes | WAIS | 51 | N/A | N/A | N/A | N/A |
| P30 | M | W | No | 25.43 | N/A | N/A | No | No | WAIS | 100 | 108 | 4 | Non-spectrum | No |
| P31 | F | W | No | 28.47 | Yes | Yes | No | Yes | WASI | 51 | 50 | N/A | N/A | N/A |
| P32 | F | W | No | 30.52 | N/A | No | No | Yes | WASI | 59 | 40 | 4 | Autism spectrum | No |
| P33 | M | W | No | 32.78 | No | No | No | Yes | WAIS | 64 | 88 | 4 | Nonspectrum | No |

**Table S3. Test statistics and exact p-values from regression models predicting diagnostic outcomes, adjusting for age.** Slopes are reproduced from Table 3 of main text, with addition of test statistic (Z-statistic for diagnostic outcome and t-statistic for continuous outcome) and unadjusted p-value. ASD Diagnosis = clinical judgment of autism spectrum disorder (yes/no; see Figure S1); ABC = Vineland Adaptive Behavior Composite; FSIQ = Full scale intelligence quotient; ID Diagnosis = clinical judgment of intellectual disability (yes/no); LOF = loss-of-function.

| Outcome | Biomarker | Slope Estimate or Odds Ratio | 95% CI Lower Bound | 95% CI Upper Bound | Test Statistic (t for FSIQ and ABC, Z for diagnosis) | p-value |
| --- | --- | --- | --- | --- | --- | --- |
| ASD Diagnosis | Erythropoietin, serum, mIU/mL | 28.112 | 2.550 | 1050.718 | 2.271 | **0.023** |
|  | FGF21, plasma, pg/mL | 0.789 | 0.432 | 1.378 | -0.825 | 0.409 |
|  | GDF15, plasma, pg/mL | 1.514 | 0.616 | 3.994 | 0.890 | 0.373 |
|  | Gene affected (*PCCA* or *PCCB*) | 0.26 | 0.05 | 1.18 | -1.71 | 0.087 |
|  | Glycine, plasma, µmol/L | 0.851 | 0.215 | 3.307 | -0.238 | 0.812 |
|  | Glutamine, plasma, µmol/L | 0.071 | 0.003 | 0.905 | -1.869 | **0.062** |
|  | *in vivo* Propionate oxidation, 60-min, % | 0.575 | 0.250 | 1.178 | -1.435 | 0.151 |
|  | Propionylcarnitine, plasma, µmol/L | 1.878 | 0.412 | 10.549 | 0.784 | 0.433 |
|  | Total 2-methylcitrate, plasma, nmol/L | 1.361 | 0.464 | 4.445 | 0.549 | 0.583 |
|  | Two LOF alleles | 3.85 | 0.76 | 22.83 | 1.59 | 0.11 |
|  | Glutamine, urine, nmol/mg creatinine | 0.482 | 0.097 | 2.068 | -0.962 | 0.336 |
| ABC | Erythropoietin, serum, mIU/mL | -25.119 | -43.226 | -7.013 | -2.870 | **0.009** |
|  | FGF21, plasma, pg/mL | -7.881 | -13.583 | -2.180 | -2.837 | **0.009** |
|  | GDF15, plasma, pg/mL | -15.922 | -24.276 | -7.569 | -3.911 | **0.001** |
|  | Gene affected | -4.12 | -21.49 | 13.26 | -0.49 | 0.63 |
|  | Glycine, plasma, µmol/L | 6.052 | -9.421 | 21.525 | 0.802 | 0.429 |
|  | Glutamine, plasma, µmol/L | 31.784 | 7.467 | 56.101 | 2.682 | **0.012** |
|  | *in vivo* Propionate oxidation, 60-min, % | 15.897 | 10.895 | 20.899 | 6.533 | **0.000** |
|  | Propionylcarnitine, plasma, µmol/L | -23.458 | -38.452 | -8.464 | -3.210 | **0.003** |
|  | Total 2-methylcitrate, plasma, nmol/L | -11.226 | -23.029 | 0.577 | -1.952 | 0.061 |
|  | Two LOF alleles | -18.98 | -36.40 | -1.6 | -2.24 | 0.03 |
|  | Glutamine, urine, nmol/mg creatinine | 11.229 | -5.675 | 28.133 | 1.378 | 0.182 |
| FSIQ | Erythropoietin, serum, mIU/mL | -36.771 | -52.618 | -20.924 | -4.779 | **0.000** |
|  | FGF21, plasma, pg/mL | -8.524 | -15.280 | -1.768 | -2.580 | **0.015** |
|  | GDF15, plasma, pg/mL | -22.232 | -30.013 | -14.451 | -5.843 | **0.000** |
|  | Gene affected | -0.985 | -29.1 | 9.4 | -1.05 | 0.30 |
|  | Glycine, plasma, µmol/L | 5.591 | -12.443 | 23.625 | 0.634 | 0.531 |
|  | Glutamine, plasma, µmol/L | 51.066 | 26.087 | 76.045 | 4.181 | **0.000** |
|  | *in vivo* Propionate oxidation, 60-min, % | 15.724 | 9.689 | 21.759 | 5.346 | **0.000** |
|  | Propionylcarnitine, plasma, µmol/L | -30.185 | -43.777 | -16.593 | -4.542 | **0.000** |
|  | Total 2-methylcitrate, plasma, nmol/L | -16.106 | -26.260 | -5.952 | -3.244 | **0.003** |
|  | Two LOF alleles | -19.2 | -38.9 | 0.5 | -1.99 | 0.05 |
|  | Glutamine, urine, nmol/mg creatinine | 17.884 | 3.754 | 32.015 | 2.612 | **0.015** |
| ID Diagnosis | Erythropoietin, serum, mIU/mL | 115.388 | 6.820 | 10245.564 | 2.655 | **0.008** |
|  | FGF21, plasma, pg/mL | 2.013 | 1.100 | 4.313 | 2.063 | **0.039** |
|  | GDF15, plasma, pg/mL | 59.829 | 6.594 | 2743.817 | 2.787 | **0.005** |
|  | Gene affected (*PCCA* or *PCCB*) | 5.5 | 1.21 | 30.13 | 2.12 | **0.03** |
|  | Glycine, plasma, µmol/L | 0.686 | 0.168 | 2.572 | -0.554 | 0.580 |
|  | Glutamine, plasma, µmol/L | 0.006 | 0.000 | 0.175 | -2.470 | **0.014** |
|  | *in vivo* Propionate oxidation, 60-min, % | 0.001 | 0.000 | 0.086 | -1.565 | 0.117 |
|  | Propionylcarnitine, plasma, µmol/L | 2203.056 | 32.132 | 8436433.304 | 2.633 | **0.008** |
|  | Total 2-methylcitrate, plasma, nmol/L | 91.813 | 7.414 | 7176.876 | 2.709 | **0.007** |
|  | Two LOF alleles | N/A^1^ |  |  |  |  |
|  | Glutamine, urine, nmol/mg creatinine | 0.032 | 0.001 | 0.262 | -2.574 | **0.01** |

^1^Quasi-complete separation occurred, because all participants (100% of n=10) harboring two loss-of-function alleles in either *PCCA* or *PCCB* (nonsense, deletions, and copy number variants or variants affecting canonical splice sites) were diagnosed with ID.

**Figure S1. ASD diagnostic process participant dispensation.** Of 33 participants, there was no concern of ASD for n = 10, two participants were not screened or evaluated for ASD due to time and personnel constraints, and n = 21 received the full diagnostic battery, including Autism Diagnostic Observation Schedule – Second Edition, Autism Diagnostic Interview – Revised, and DSM-5 clinical judgment. As a result, n = 12 of 31 participants (39%) were diagnosed with ASD and n = 19 of 31 (61%) were determined to be non-spectrum.


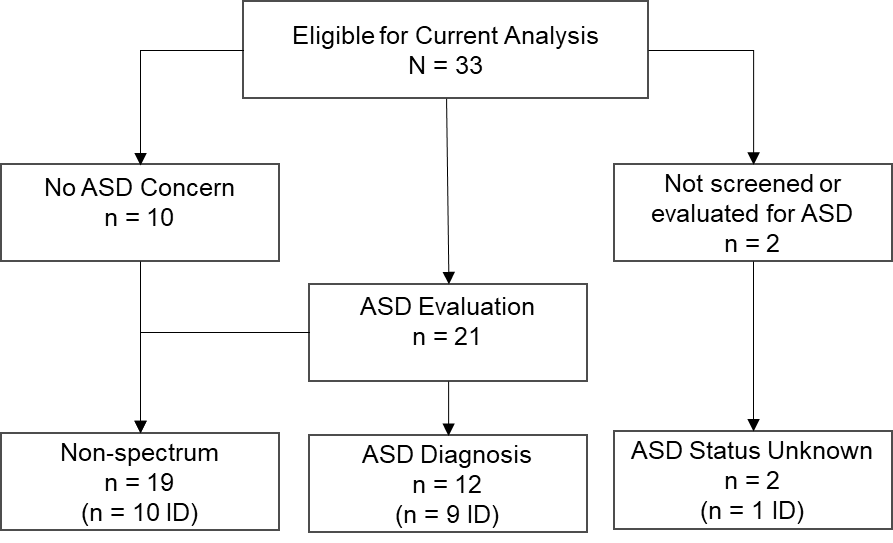


**Figure S2. Distributions of continuous variables.** ABC = Vineland Adaptive Behavior Composite; FSIQ = Full scale intelligence quotient.

. **
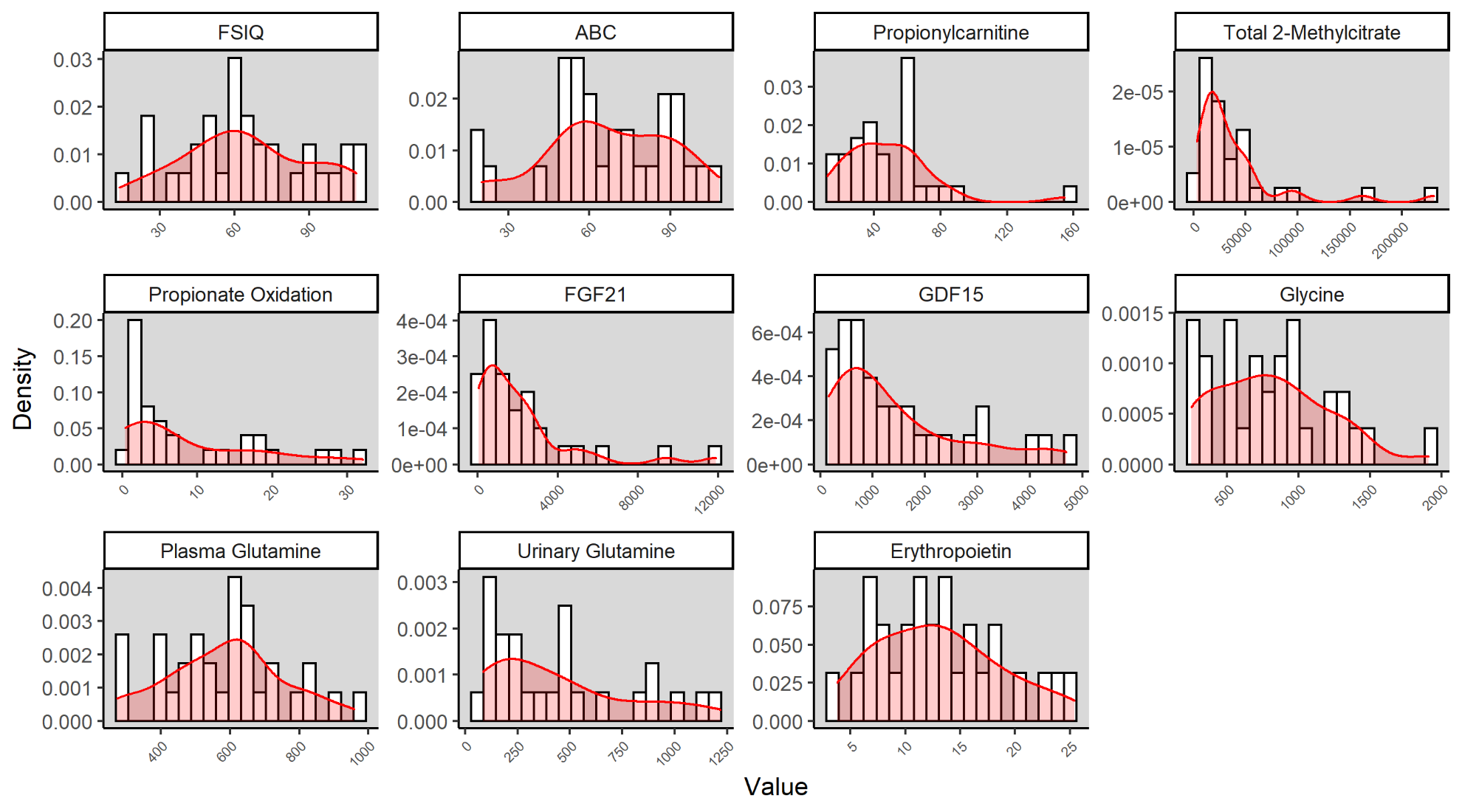
**

**Figure S3. ASD diagnosis in PA is not associated with most biomarkers linked to the mitochondrial dysfunction and the severity of PCC deficiency**. Biomarkers shown here as natural log-transformed values were linked via regression to the underlying severity of PA and mitochondrial dysfunction and showed little relationship to ASD diagnosis among individuals with PA. Orange points are individuals who received an ASD diagnosis (n=12) and purple are those who were not diagnosed with ASD (n=19). Two participants for whom ASD status was unknown were excluded. The log-transformed predictor variable for each panel is named in the header. Parameter estimates corresponding to these lines are shown in Table 3 and test statistics in Supplementary Table S3. The distributions of untransformed continuous biomarkers are shown in Supplemental Figure S2. Panel A: plasma propionylcarnitine (ln-transformed); Panel B: plasma total 2-methylcitrate (ln-transformed); Panel C: in vivo whole body 1-13C-propionate oxidation (ln-transformed); Panel D: plasma FGF21 (ln-transformed); Panel E: plasma GDF15 (ln-transformed); Panel F: plasma glycine (ln-transformed); Panel G: plasma glutamine (ln-transformed); Panel H: urinary concentration of glutamine normalized by creatinine (ln-transformed); Panel I: serum erythropoietin (ln-transformed); Panel J: affected gene, PCCA vs PCCB; Panel K: comparison of neurocognitive outcomes in participants with two (biallelic) loss-of-function PCCA or PCCB alleles vs all other genotypes (e.g. two missense alleles or one missense plus one nonsense allele).


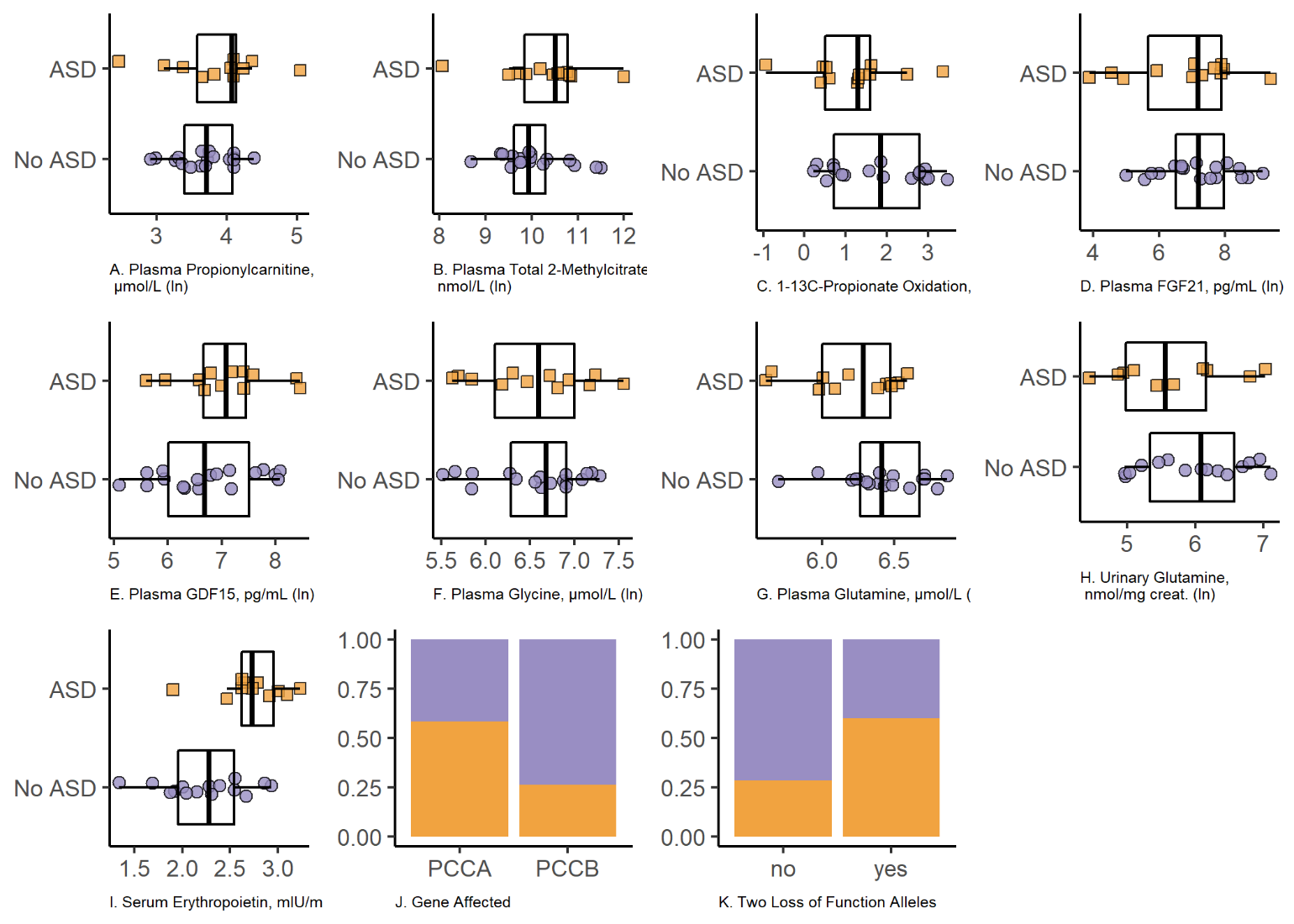

Supplement: Supplementary file 1 — Supplementary materials [file 41380_2023_2385_MOESM1_ESM.docx]
